# Time-Series Analysis and Age-Stratified Forecasting of Diarrheal Disease in Rwanda Using SARIMA Models

**DOI:** 10.3390/tropicalmed11050130

**Published:** 2026-05-11

**Authors:** Theos Dieudonne Benimana, Martin Habimana, Jean de Dieu Harerimana, Eric Mugabo, Thierry Sebakunzi, Patrick Niyonshuti, Valens Rwema, Muhammed Semakula, Seung-sik Hwang

**Affiliations:** 1 National Health Intelligence Center, Ministry of Health, Kigali P.O. Box 84, Rwanda; dieudonne.benimana@moh.gov.rw (T.D.B.); eric.mugabo@moh.gov.rw (E.M.); sebakunzithierry@gmail.com (T.S.); patrick.niyonshuti@moh.gov.rw (P.N.); valens.rwema@moh.gov.rw (V.R.); 2 Department of Public Health, Seoul National University, Seoul 08826, Republic of Korea; 3 Centre for Financial Regulation and Inclusion (CENFRI), Kigali P.O. Box 1599, Rwanda; martinhabiman@gmail.com; 4 Clinton Health Access Initiative, Kigali P.O. Box 6169, Rwanda; jharerimana@clintonhealthaccess.org; 5 African Centre of Excellence in Data Science, University of Rwanda, Kigali P.O. Box 1514, Rwanda; semakulam@gmail.com

**Keywords:** diarrhea, forecasting, SARIMA, time series, seasonal variation, Rwanda

## Abstract

Background: Diarrheal disease remains a major and persistent cause of morbidity and mortality in Rwanda, with substantial seasonal surges that strain routine services; however, transparent and operationally interpretable national forecasting has been underused for age-stratified burden. Methods: We analyzed the Rwanda Health Management Information System (HMIS) monthly diarrhea case counts (January 2015–December 2025), stratified by age group (under-five and five-and-above), and developed validated Seasonal Autoregressive Integrated Moving Average (SARIMA) forecasts for January 2026–December 2027. Stationarity was assessed using the Augmented Dickey–Fuller test and addressed through differencing. Candidate models were selected via rolling 5-fold cross-validation: Root Mean Square Error (RMSE), Mean Absolute Error (MAE), Akaike Information Criterion (AIC), Bayesian Information Criterion (BIC), and Mean Absolute Percentage Error (MAPE) and confirmed via Ljung–Box residual diagnostics, and benchmarked against seasonal naïve, Exponential Smoothing State-Space (ETS), and Seasonal-Trend decomposition using Loess (STL) + drift reference models. Results: Rwanda recorded 6,309,098 diarrhea cases during 2015–2025, with 49.2% among under-fives; while absolute counts were higher in those aged ≥5 years, risk remained consistently higher in under-fives (91.7–229.5 per 1000) than in those ≥5 years (17.9–34.3 per 1000). Both series showed strong annual seasonality with recurrent peaks in August–November, and forecasts suggest this pattern will persist through 2026–2027. Conclusions: These findings suggest a provisional seasonal (pre-peak, peak, and post-peak) preparedness framework and age-differentiated planning signals, underscoring that burden and risk are not inter changeable across age groups.

## 1. Introduction

Diarrheal diseases continue to constitute a major global public health challenge, particularly among children under five years of age, where they remain the third leading cause of mortality [1]. Globally, an estimated 1.7 billion cases of childhood diarrhea occur annually, resulting in approximately 443,832 deaths in children under five and an additional 50,851 deaths in children aged five to nine years [1]. The burden is disproportionately higher in low- and middle-income countries, particularly in sub-Saharan Africa, where limited access to safe drinking water, poor sanitation, and inadequate hygiene practices significantly contribute to both incidence and mortality [2,3,4].

Within the African region, diarrheal diseases remain a pressing concern. A recent systematic review and meta-analysis estimated approximately 1.008 billion diarrhea cases and 515,031 associated deaths in 2020, with children under five years being the most affected group. Eastern Africa recorded the highest incidence in this age group, while Western Africa experienced the highest mortality [2,5].

In Rwanda, diarrheal diseases remain a leading cause of childhood morbidity and mortality, especially among children under five. The diarrhea prevalence increased from 12.2% (931 cases out of 7627 sampled children) in the 2014/15 Rwanda Demographic and Health Survey (RDHS) [6] to 14.3% (1141 cases out of 7974 sampled children) in the 2019/20 RDHS [7], and further to 15% in the 2025 RDHS [8]. This persistent upward trajectory underscores a continuing public health challenge influenced by various sociodemographic determinants. Children aged 12 to 23 months are particularly vulnerable, and risk is elevated among children from lower socioeconomic statuses. Geographic disparities are also evident, with the Western Province exhibiting higher prevalence rates. High maternal education levels appear to be protective, as children of less educated mothers are disproportionately affected. Moreover, engagement in agricultural labor has been linked to increased diarrhea incidence through several pathways: environmental exposure to pathogens via contact with soil, livestock, and surface water; limited access to improved water and sanitation in rural agricultural communities; greater socio-economic vulnerability among smallholder farming households, which constrains Water, Sanitation, and Hygiene (WASH) investment; and reduced supervision of young children when caregiver time is diverted to field activities [3,9].

Effective preventive measures are well-established and include improved sanitation, safe water access, handwashing with soap, exclusive breastfeeding for the first six months, and rotavirus vaccination; oral rehydration therapy remains the proven treatment. Despite this, care-seeking behavior for diarrheal illness has stagnated in Rwanda. Between 2014/15 and 2019/20, the proportion of children under five who were taken for treatment remained nearly unchanged (53% vs. 52%, respectively) [7]. This lack of improvement, coupled with the rising incidence of diarrhea, highlights an urgent need for renewed policy attention and resource allocation to tackle diarrheal disease more effectively. Furthermore, as Rwanda navigates the dual burden of infectious and non-communicable diseases, there is growing concern that infectious diseases, such as diarrhea, may receive diminishing focus, despite their continued high burden and preventability.

Accurate forecasting of disease burden using time-series models supports evidence-based public health planning and timely resource allocation. SARIMA models have demonstrated particular utility across diverse sectors, including energy, economics, agriculture, and demography [10,11,12,13,14,15], and have been widely applied in health to forecast both infectious and non-infectious disease trends [16,17,18,19,20,21,22,23,24,25,26,27,28]. Previous predictive studies of diarrheal disease have applied several distinct families of models. Regression-based and multilevel models have primarily focused on identifying socioeconomic and environmental determinants of childhood diarrhea [3,9,29,30,31,32] rather than forecasting future incidence, limiting their operational value for preparedness and early warning.

More recently, machine-learning and deep-learning approaches, including random forest, gradient boosting, Long Short-Term Memory (LSTM), and ensemble models, have been increasingly applied to diarrheal disease prediction and forecasting [33,34,35,36,37,38,39], often demonstrating improved accuracy when rich covariates such as meteorological or syndromic surveillance data are available. However, as highlighted by the recent systematic review of predictive modeling for infectious diarrheal disease, inconsistent validation practices, high data requirements, and limited interpretability constrain the reproducibility and routine adoption of these models within national surveillance systems [40].

In contrast, classical time-series models, particularly Autoregressive Integrated Moving Average (ARIMA) and its seasonal extension SARIMA, have been less emphasized in diarrhea research despite their established role in infectious disease surveillance and forecasting. SARIMA provides a concise and interpretable framework that explicitly models temporal autocorrelation and seasonality using routinely collected case counts alone, without reliance on external covariates that may be unavailable or inconsistently reported [19,28,41,42,43]. Its selection for this study reflects three operational realities. First, no climate, environmental, or socioeconomic covariates are consistently available at the national monthly level in Rwanda’s HMIS, limiting the applicability of covariate-dependent approaches that outperform SARIMA specifically when covariate data are integrated [43]. Second, the 132-month dataset suits SARIMA’s parameter efficiency but is insufficient for deep learning architectures that require substantially larger series. Third, hybrid models consistently build on SARIMA as the essential linear seasonal component rather than replacing it [41,42], confirming its role as the appropriate baseline. The systematic review of predictive modeling for infectious diarrheal disease further confirms that inconsistent validation practices and high data requirements constrain the routine adoption of more complex models within national surveillance systems [40]. Seasonal AutoRegressive Integrated Moving Average with eXogenous regressors (SARIMAX) and hybrid extensions are explicitly identified as the designated next step.

The aim of this study is therefore to (1) characterize the temporal trend and seasonal structure of diarrheal morbidity by age group; (2) develop and validate age-stratified SARIMA models; (3) generate short- to medium-term forecasts to inform seasonal preparedness planning; and (4) establish a transparent, reproducible baseline framework suitable for integration into Rwanda’s routine HMIS surveillance workflow.

## 2. Materials and Methods

### 2.1. Study Design and Setting

This study employed an ecological retrospective observational design using routinely collected national health data to forecast monthly diarrhea cases in Rwanda. The study aimed to generate prospective forecasts for January 2026 to December 2027 based on historical data from January 2015 to December 2025. The setting included all public and private health facilities that report to the Rwandan Health Management Information System (HMIS), ensuring comprehensive national coverage. Data were stratified into two age groups: children under five years and individuals aged five years and above. This stratification reflects three considerations. First, children under five bear a well-documented disproportionate burden of diarrheal incidence and case fatality [1,2,30]. Second, Rwanda’s HMIS records diarrhea cases by these exact age strata at the facility level, ensuring alignment with reporting architecture. Third, preliminary inspection revealed distinct temporal dynamics between the two series, justifying separate SARIMA model specifications.

### 2.2. Participants and Data Sources

The study utilized aggregated monthly counts of reported diarrhea cases from Rwanda’s HMIS: https://aggregate.moh.gov.rw (accessed on 7 January 2016), spanning January 2015 to December 2025, yielding 132 monthly observations per age group. Data entry is carried out at the facility level and routinely audited for completeness and accuracy by district health information officers. Inclusion criteria comprised all age-specific records with complete monthly reporting between 2015 and 2025. Population estimates were obtained from the National Institute of Statistics of Rwanda (NISR) Fifth Rwanda Population and Housing Census (RPHC5) [44]. Variables extracted included month and year of report, age group (<5 years or ≥5 years), and number of diarrhea cases per month.

### 2.3. Case Definition and Outcome Measure

A diarrhea case was defined following Rwandan Ministry of Health and World Health Organization standards as the passage of three or more loose or liquid stools within a 24-h period [45]. Both outpatient and inpatient cases were included to ensure a comprehensive view of the national burden. The primary outcome was the monthly count of diarrhea cases, disaggregated by age group. These counts were treated as a univariate time series in the forecasting model. Throughout this study, absolute monthly case counts serve as both the modeling input and the primary outcome for forecasting. Incidence rates (per 1000 population) are reported descriptively solely to enable age-specific risk comparison across population groups of different sizes and are not used in the forecasting models.

### 2.4. Statistical Methods: Forecasting and Evaluation

#### 2.4.1. Model Specification

Monthly diarrheal case counts were modeled using the Seasonal Autoregressive Integrated Moving Average (SARIMA) framework, denoted as follows:

(1) $$SARIMA\left(p,d,q\right){\left(P,D,Q\right)}_{s}$$

where p,d,q are the non-seasonal autoregressive (AR), differencing, and moving-average (MA) orders; P,D,Q are the corresponding seasonal orders; and s is the seasonal period. For monthly observations s=12. Following the Box–Jenkins formulation [46,47], let B be the backshift operator, such that $B^{k}y_{t}=y_{t-k}$.

The general SARIMA model is expressed as follows:

(2) $$\Phi \left(B^{s}\right)\phi \left(B\right){\left(1-B\right)}^{d}{\left(1-B^{s}\right)}^{D}y_{t}=\Theta \left(B^{s}\right)\theta \left(B\right)\varepsilon_{t}$$

where $\varepsilon_{t}$ is a zero-mean white noise innovation with constant variance. The model polynomials are defined as follows:

Non-seasonal AR polynomial: $\phi \left(B\right)=1-\phi_{1}B-\cdots -\phi_{p}B^{p}$

Non-seasonal MA polynomial: $\theta \left(B\right)=1-\theta_{1}B-\cdots -\theta_{q}B^{q}$

Seasonal AR polynomial (period s): $\Phi \left(B^{s}\right)=1-\Phi_{1}B^{s}-\cdots -\Phi_{P}B^{Ps}$

Seasonal MA polynomial (period s): $\Theta \left(B^{s}\right)=1-\Theta_{1}B^{s}-\cdots -\Theta_{Q}B^{Qs}$

Non-seasonal and seasonal differencing are represented by ${\left(1-B\right)}^{d}$ and ${\left(1-B^{s}\right)}^{D}$, respectively.

#### 2.4.2. Stationarity Assessment and Model Selection

Stationarity was evaluated using the Augmented Dickey–Fuller (ADF) test [48]:

(3) $$\Delta y_{t}=\alpha +\beta t+\gamma y_{t-1}+\sum_{i=1}^{k}\delta_{i}\Delta y_{t-i}+\varepsilon_{t}$$

testing $H_{0}:\gamma =0$ (unit root) against $H_{1}:\gamma <0$ (stationary). When evidence of non-stationarity was observed (p>0.05), differencing was applied via ${\left(1-B\right)}^{d}$ and/or ${\left(1-B^{s}\right)}^{D}$ until stationarity was supported. Candidate SARIMA models were explored via systematic grid search over non-seasonal orders $p,q\in \left\{0,1,2,3\right\}$ and d=1 (confirmed using ADF tests) and seasonal orders $P,Q\in \left\{0,1\right\}$ $D\in \left\{0,1\right\}$. Model selection was guided by rolling-origin time-series cross-validation within the training set (January 2015–September 2023; 105 months). The series was partitioned into up to five sequential validation blocks, each covering 21 months (20% of training length). For each fold, the model was fit using all observations preceding the validation block and evaluated by forecasting the subsequent 21 months. Validation periods were strictly after their corresponding training periods, with no gap introduced, preventing information leakage. Folds with insufficient training history were not evaluated. The final model was selected based on minimum cross-validated RMSE, with AIC and BIC serving as secondary tie-breaking criteria. In both age groups, the Cross-Validation (CV)-RMSE-optimal model also yielded competitive AIC and BIC values, eliminating ambiguity in the final selection [46,47]. To assess the incremental predictive value of the selected SARIMA specifications beyond naïve seasonal repetition or trend extrapolation, the final models were additionally benchmarked against three reference forecasting approaches: a seasonal naïve model, an automatically selected Exponential Smoothing State-Space model (ETS), and a Seasonal-Trend decomposition using Loess with a random-walk-with-drift forecaster on the seasonally adjusted component (STL + drift). Each benchmark model was fit on the identical training partition (January 2015–September 2023) and was evaluated on the same 27-month hold-out test set (October 2023–December 2025) used for SARIMA test-set evaluation, using the metrics defined below.

#### 2.4.3. Forecasting and Evaluation Metrics

The final SARIMA model was refitted on the full dataset (January 2015–December 2025) and used to forecast monthly cases from January 2026 to December 2027. The accuracy of short-term predictions was assessed using a test set comprising October 2023–December 2025 (27 months), and the following performance metrics were calculated:

(4) $$\mathrm{Root}\;\mathrm{Mean}\;\mathrm{Square}\;\mathrm{Error}\;(\mathrm{RMSE})\;=\sqrt{\frac{1}{n}\sum_{t=1}^{n}{\left(y_{t}-{\hat{y}}_{t}\right)}^{2}}$$

(5) $$\mathrm{Mean}\;\mathrm{Absolute}\;\mathrm{Error}\;(\mathrm{MAE})=\frac{1}{n}\sum_{t=1}^{n}{|y}_{t}-{\hat{y}}_{t}|$$

(6) $$\mathrm{Mean}\;\mathrm{Absolute}\;\mathrm{Percentage}\;\mathrm{Error}\;(\mathrm{MAPE})=\frac{100}{n}\sum_{t=1}^{n}\left|\frac{y_{t}-{\hat{y}}_{t}}{y_{t}}\right|$$

Although MAPE is undefined when actual values approach zero, this is not a concern in the present study. The minimum observed monthly case count across the full study period is 10,585 for children under five (February 2015) and 9510 for individuals aged five and above (August 2016), confirming that near-zero observations are not possible in this surveillance context.

Residual autocorrelation was assessed using the Ljung–Box test [49]. Model adequacy was concluded when residuals were consistent with white noise and no systematic structure remained in the residual autocorrelation function.

All analyses were performed in R (version 4.5.2; R Foundation for Statistical Computing, Vienna, Austria) using RStudio (Version 2026.01.0+392; Posit Software, PBC, Boston, MA, USA). We used the ‘forecast’ package (version 9.0.1) for SARIMA model fitting, forecasting, and benchmark model construction (seasonal naïve, ETS, and STL + drift); ‘tseries’ (version 0.10.60) for the ADF test; and the base ‘stats’ package (included with R) for Ljung–Box residual diagnostics.

### 2.5. Ethical Consideration

Ethical review and approval were waived for this study, as it utilized secondary data from Rwanda’s HMIS, which is anonymized and aggregated at the facility level, ensuring no personal identifiers were accessible. Permission to access and analyze this data was granted to the National Health Intelligence Center (NHIC) under the Ministry of Health. All procedures adhered to the ethical principles outlined in the Declaration of Helsinki.

## 3. Results

### 3.1. Descriptive Epidemiology of Diarrhea Cases (2015–2025)

#### 3.1.1. Total Burden of Diarrhea (2015–2025)

Rwanda reported 6,309,098 diarrhea cases between 2015 and 2025, with nearly half (49.2%) occurring among children under five years of age. Although annual cases more than doubled in both age groups over the study period, age-specific population estimates increased over time. Age-specific incidence rates reveal a persistent and disproportionate burden among children under five years, whose incidence remained consistently several-fold higher than that of individuals aged five years and above. Under-five incidence ranged from 91.7 to 229.5 cases per 1000 population per year, compared with 17.9 to 34.3 per 1000 among those aged five years and above.

#### 3.1.2. Annual and Monthly Case Trends

**Figure 1:**
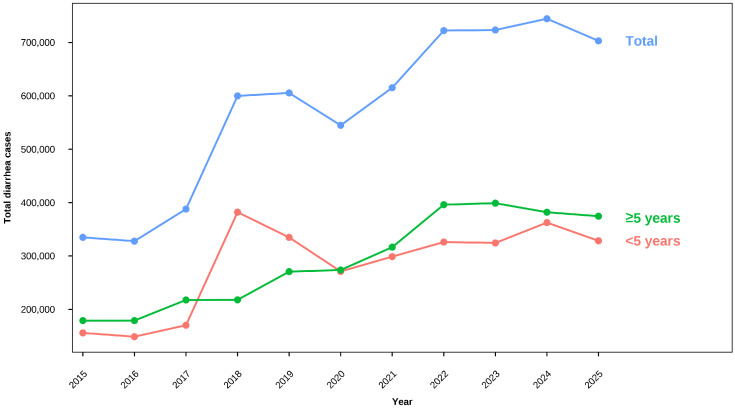
Yearly diarrhea case trends by age category, 2015–2025.

Figure 1 shows an overall increase in reported diarrhea cases in Rwanda during the study period, with notable year-to-year fluctuations. Although absolute case counts are higher among individuals aged five and above, particularly after 2019, incidence rates remain consistently higher among children under five, as shown in Table 1. A sharp surge in under-five cases in 2018 was followed by a temporary decline and subsequent resurgence, while cases among older individuals increased more gradually over time. Under-five diarrhea cases declined by 19.0% between 2019 and 2020 (from 334,801 to 271,043), coinciding with COVID-19-related reductions in health-seeking behavior and outpatient facility utilization following national lockdown measures implemented from March 2020 [50]. Cases rebounded in 2021 (298,797), with case counts remaining elevated and variable through 2022–2025.

**Figure 2:**
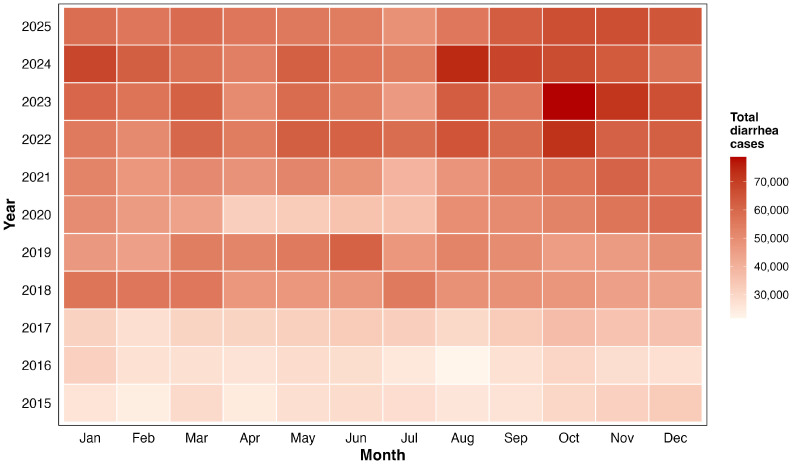
Calendar heatmap of monthly diarrhea case counts by year, 2015–2025.

Figure 2 presents a calendar heatmap of monthly diarrhea case counts across all study years (2015–2025), with color intensity proportional to case burden. The heatmap reveals a strong, recurrent seasonal pattern: cells are consistently lighter from January to July, with progressive darkening toward August–November, confirming a stable peak period aligned with Rwanda’s dry-to-short-rains seasonal transition. This signal is reproducible across all 11 study years. Beyond seasonality, the heatmap shows a clear upward interannual trend, with rows progressively darkening from 2015 to 2022–2025, reflecting rising baseline and peak burden. Episodic high-intensity cells in October 2023 and August 2024 represent the highest monthly case counts of the study period. Notably, the 2020 row appears visibly lighter across most months, consistent with COVID-19-related reductions in health facility utilization rather than a genuine decline in transmission.

### 3.2. Modeling and Forecasting of Diarrheal Disease Cases Using SARIMA

#### 3.2.1. Final SARIMA Model Specification, Diagnostics, and Interpretation

This study applied a SARIMA model to forecast diarrhea cases in Rwanda across two age groups: children under five years and individuals aged five years and above, using monthly HMIS data from January 2015 to December 2025. Prior to model fitting, stationarity was assessed using the Augmented Dickey–Fuller (ADF) test. For the under-five series, the original series was non-stationary (ADF = –2.024, *p* = 0.566), while the first-differenced series achieved stationarity (ADF = −5.503, *p* = 0.01). Similarly, for individuals aged five years and above, the original series was non-stationary (ADF = −3.246, *p* = 0.083) and became stationary after first differencing (ADF = −4.992, *p* = 0.01), supporting the use of d = 1 in both age-stratified models.

Table 2 summarizes the final SARIMA specifications selected via rolling time-series cross-validation, alongside key fit and forecast performance metrics. For children under five years, the selected model was SARIMA(2,1,0)(1,0,1)[12] (lowest cross-validated RMSE), while for individuals aged five years and above, the optimal model was SARIMA(1,1,0)(1,0,1)[12]. Parameter estimates for both models are presented in Table 3.

Both age-specific time series showed pronounced annual seasonality (s = 12). Across both final models, residual diagnostics supported adequate fit: residuals were centered around zero with no systematic structure, the residual autocorrelation function was largely within 95% bounds, and Ljung–Box tests provided no evidence of remaining serial correlation (*p* > 0.05).

Table 3 presents the full parameter estimates for both final models. For children under five, all four model coefficients were statistically significant. The AR(2) structure reflects a momentum-and-correction dynamic (AR1 = 0.868, *p* < 0.001; AR2 = −0.442, *p* < 0.001). Diarrheal burden strongly tracks the preceding month before partially self-correcting over a two-month window, consistent with the episodic surge-and-recovery patterns observed in 2018 and 2020–2021. The seasonal autoregressive term (SAR1 = 0.768, *p* < 0.001) confirms strong year-on-year seasonal persistence as the dominant temporal driver, and the seasonal moving average term (SMA1 = −0.531, *p* = 0.026) indicates that unusually large seasonal shocks are partially corrected in the following annual cycle.

For individuals aged five years and above, all three model parameters were statistically significant. The near-unit seasonal autoregressive term (SAR1 = 0.925, *p* < 0.001) indicates near-complete year-on-year persistence in month-specific burden, more stable than the under-five series (SAR1 = 0.768), reflecting the absence of episodic transmission surges of the magnitude observed in young children. The seasonal moving average term (SMA1 = −0.650, *p* < 0.001) captures strong correction of seasonal shocks, and the mild negative non-seasonal term (AR1 = –0.216, *p* = 0.012) indicates slight mean reversion between consecutive months.

To demonstrate that the selected SARIMA specifications capture temporal structure beyond simple seasonal repetition or trend extrapolation, the final models were benchmarked against three reference forecasting approaches on the identical 5-fold rolling-origin cross-validation procedure: (i) the seasonal naïve model, which constitutes the canonical baseline for seasonal time series; (ii) an automatically selected exponential smoothing state-space model (ETS), with model variant chosen by AICc minimization across all admissible ETS specifications; and (iii) STL decomposition with a random-walk-with-drift forecaster on the seasonally adjusted component (STL + drift), a stronger trend-aware benchmark that explicitly separates trend, seasonal, and remainder components.

For the under-five group, SARIMA(2,1,0)(1,0,1)[12] achieved the lowest cross-validated MAPE (6.7%) of all models evaluated, representing MAPE reductions of 68.2%, 57.4%, and 57.2% relative to the seasonal naïve, ETS, and STL + drift benchmarks, respectively. For the five-and-above group, SARIMA(1,1,0)(1,0,1)[12] achieved the lowest RMSE and MAE. While STL + drift yielded a marginally lower MAPE in this stratum (7.55% vs. 9.1%), SARIMA produced a substantially lower RMSE (2187 vs. 2320), indicating superior performance on large-magnitude forecast errors, the operationally more consequential metric for health service surge planning, where underestimating peak burdens carries greater programmatic risk than overestimating routine demand. Critically, SARIMA was not selected in isolation on a single metric for a single stratum.

The selection rationale integrates four considerations that collectively support SARIMA over STL + drift as the operational framework: (1) SARIMA achieves the lowest RMSE in both age strata, while STL + drift achieves the lowest MAPE in only one; (2) SARIMA yields interpretable autoregressive and seasonal parameters with formal statistical inference (Table 3), enabling substantive epidemiological interpretation of temporal dynamics; (3) SARIMA provides a formal probabilistic framework for prediction interval generation, which is essential for exceedance-based preparedness planning and cannot be natively obtained from STL + drift without additional assumptions; and (4) the seasonal naïve and ETS benchmarks, both clearly outperformed by SARIMA across all metrics, confirm that the improvements are not an artifact of the comparison set.

#### 3.2.2. Modeling and Forecasting

**Figure 3:**
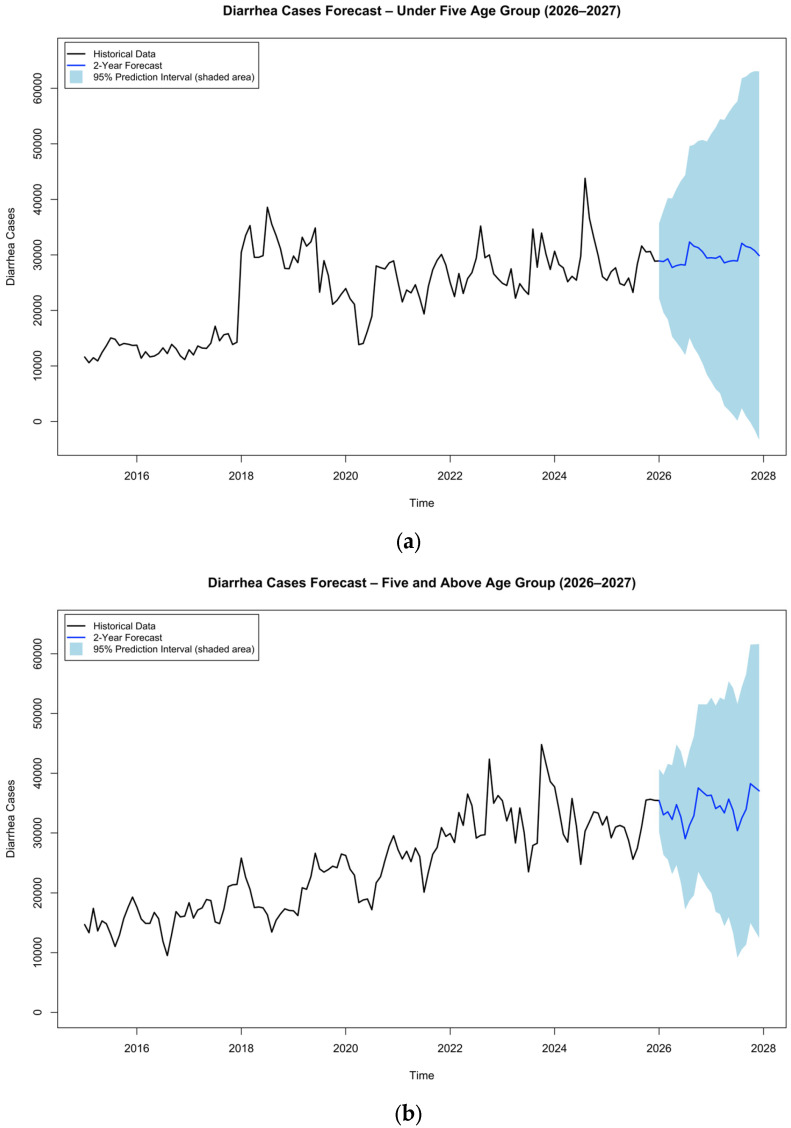
(**a**) Diarrhea cases forecast for children under five. (**b**) Diarrhea cases forecast for individuals aged five and above. The blue shaded area in (**a**,**b**) represents the 95% prediction interval (PI) of the SARIMA forecast, with bounds widening progressively with forecast horizon.

Figure 3 displays that during the forecast period (2026–2027), monthly diarrhea cases are projected to remain relatively stable in both age groups. Children under five average about 29,747 cases per month, whereas those aged five and above average around 34,298 cases per month (Table 4). The forecasted trends in both groups exhibit pronounced seasonality, with peaks consistently expected between August and November, mirroring historical seasonal patterns. This regularity highlights the persistence of late-dry-season surges in diarrhea case burden across all age categories. Although point forecasts change modestly, forecast uncertainty increases with horizon: 95% prediction intervals widen progressively, most notably from late 2026 onward, reflecting the reduced precision of longer-range forecasts from routine surveillance time series.

SARIMA forecasts for 2026–2027 in Table 4 indicate that across both age groups, there is no major shift between 2026 and 2027; diarrhea will likely remain an endemic, recurrent burden requiring sustained prevention and response capacity rather than short-term surge-only measures. Although individuals aged five and above are projected to contribute higher annual case totals on average across the forecast period (411,570 vs. 356,957 for under-fives), this largely reflects their larger population size rather than elevated per-child risk. Children under five remain the highest-risk group on a per-child basis, consistent with historical patterns. Consistent with patterns observed from 2015 to 2025, both age groups are expected to experience pronounced spikes between August and November, underscoring the persistence of late-dry-season peaks. Finally, the wider 95% prediction intervals in 2027 indicate declining precision with increasing forecast horizon; therefore, longer-range estimates should be treated as scenario guidance and updated iteratively as new surveillance data become available.

## 4. Discussion

Rwanda has experienced a sustained and increasing diarrheal burden over the past decade [6,7,8]. This upward trajectory is driven by a complex interplay of risk factors, including young child age (highest vulnerability at 12–23 months), low household wealth index, low maternal education, Western Province residence, unimproved water and sanitation facilities, unimproved toilet facilities, incomplete immunization status, bottle feeding, and engagement in agricultural labor [3,9,51].

The increased diarrheal burden from the RDHS series is in line with the clear upward trajectory in total reported diarrhea cases across both age groups over the study period, with combined annual totals doubling from 334,865 in 2015 to a peak of 703,004 in 2025. While incidence rates remained consistently higher among children under five throughout, the five-and-above group contributed a growing share of absolute caseload from 2019 onward, reflecting both population growth and rising baseline transmission in this age group. A notable exception was 2020, when under-five cases declined by 19.0% (from 334,801 to 271,043), consistent with COVID-19-related reductions in health-seeking behavior following national lockdown measures from March 2020 [50]. This decline is interpreted as a utilization artifact rather than a genuine reduction in transmission. The rapid rebound to 298,797 cases in 2021 and subsequent escalation support this interpretation. Marked surges in both absolute case counts and age-specific incidence rates in 2018 and 2022 may reflect changes in transmission dynamics, environmental conditions, or reporting practices. The growing contribution of the five-and-above group from 2019 onward reflects population size effects rather than a reversal of age-specific risk, underscoring the need for child-prioritized prevention alongside age-inclusive service readiness.

The SARIMA model results and forecast outputs further corroborate the seasonal structure revealed in Figure 2. The stable August–November peak visible across all 11 years of the heatmap is reproduced in both final SARIMA models and forecasted horizons forward through 2026–2027, confirming that this seasonal signal is sufficiently consistent to support short-term forecasting. Epidemiologically, this recurrent peak is consistent with evidence that diarrheal dynamics are sensitive to hydroclimatic variability and environmentally mediated fecal–oral transmission pathways, including water quality deterioration and contamination following rainfall events [52,53,54,55,56]. In Rwanda, the peak timing aligns with the end of the long dry season (June to mid-September) and the onset of the short rains (mid-September to mid-December). During this transition, intermittent water scarcity can increase reliance on unimproved sources, while first-flush runoff mobilizes pathogens into community water supplies, jointly elevating exposure risk. We note that this hydroclimatic interpretation is drawn from biological plausibility and prior literature rather than formal correlation analysis within this study, and formal integration of climate covariates is identified as a priority next step.

The progressive darkening of the heatmap rows and the episodic high-intensity cells in October 2023 and August 2024 further suggest that while seasonal timing is stable and predictable, peak magnitude varies year-on-year, reinforcing the operational case for rolling short-term forecasts updated as new surveillance data become available. Notably, the SARIMA model reproduced these dynamics in both the historical series and the forecast horizon, capturing distinct seasonal oscillations with stable peak timing across years, an empirical pattern consistent with other SARIMA-based infectious disease forecasting studies showing persistent seasonality and predictable peak windows [41,42,57,58,59]. The stable seasonal peak window, rising interannual baseline, and episodic surge months together indicate that static preparedness thresholds from earlier study years will systematically underestimate current and future demand. Rolling short-term forecasts, updated as new surveillance data become available, are therefore operationally necessary.

Of particular importance is the age-stratified analysis. The age-stratified forecasts provide two operational signals for Rwanda’s health system. First, while individuals aged five years and above contribute a substantial caseload and service workload, the growing numerical contribution of older age groups to total reported cases reflects population size rather than elevated age-specific risk. Under-five children remain the most vulnerable group, consistent with evidence from sub-Saharan Africa, where children in this age group, particularly those aged 12–23 months, continue to experience the highest diarrhea prevalence [30,60]. This distinction is programmatically critical: prevention and risk-reduction interventions must remain child-centered, while service readiness is maintained age-inclusive to match the full caseload profile.

Second, forecast uncertainty widens with increasing forecast time, reinforcing that SARIMA is most reliable for near-term planning. This pattern is consistent with prior epidemic forecasting work, including during the COVID-19 pandemic, where predictive performance deteriorated with longer forecast horizons [61,62]. The 95% PI does not bound structural breaks from unmodeled exogenous covariates, which fall outside the model’s seasonal structure. The upper PI bound is the more operationally relevant quantity, representing the plausible high-demand scenario, and can inform commodity stockpiling and surge capacity planning without overcommitting resources to the central point forecast. The widening intervals in 2027 reflect the standard compounding of forecast variance over longer horizons; 2026 forecasts should be treated as relatively reliable near-term signals and 2027 forecasts as indicative scenario guidance, with both updated on a rolling basis as new HMIS data become available.

To address this limitation, future applications may benefit from extending the current framework to SARIMAX, incorporating Rwanda Meteorology Agency monthly rainfall data as a candidate exogenous variable, given the hydroclimatic basis of the August–November seasonal peak. Alternatively, hybridizing SARIMA with gradient boosting or random forest models that have demonstrated improved predictive performance in related public health applications represents a promising avenue. The current models achieve CV MAPE of 6.7% (under-five) and 9.1% (five-and-above), superior to the seasonal naïve, ETS, and STL + drift benchmarks across most metrics (Table 2), representing the precision ceiling of a univariate approach; hybrid or covariate-informed models would be expected to reduce forecast error, particularly during episodic surge years and COVID-19-related reporting disruptions that fall outside the model’s seasonal structure.

These forecasts suggest a provisional seasonal preparedness framework. Pre-peak months (June–July) represent the optimal window for commodity pre-positioning (Oral Rehydration Salts (ORS), zinc) and community messaging. The August–November peak period calls for surge response protocols, and post-peak months (November–December) offer the opportunity for after-action review and rolling forecast updates. Exceedance of the upper prediction interval bound or consecutive months above a seasonal baseline may indicate a need for targeted responses, including Water, Sanitation, and Hygiene (WASH) campaigns, school-based hygiene reinforcement, and intensified water safety checks in high-burden catchments. Although SARIMA is not causal, it provides a transparent, reproducible benchmark for decision support [46,47], aligned with guidance emphasizing ORS plus zinc as the core childhood diarrhea management package [63,64,65,66].

Key limitations affect how these insights should be applied in Rwanda. The model does not incorporate exogenous drivers such as climate- and environmental-related factors, water supply disruptions, or policy and reporting practices. This can induce structural breaks and bias forecasts, constituting a form of omitted variable bias whereby excluded drivers correlated with diarrheal transmission are absorbed into the residual structure and misattributed to the seasonal signal. Climate and environmental factors directly influence pathogen mobilization. Their omission means the model cannot distinguish hydroclimatic forcing from interannual variation in transmission intensity. As a result, the burden may be underestimated during high-rainfall seasons and overestimated during dry seasons. Changes in public health interventions may progressively reduce true incidence or alter care-seeking behavior in ways the model cannot detect, causing systematic overestimation of future caseload; conversely, improvements in HMIS reporting completeness could be misinterpreted as genuine incidence increases [67]. These limitations underscore that SARIMA forecasts should be treated as pattern-based baselines rather than causal projections, interpreted alongside contextual knowledge of ongoing interventions and environmental conditions.

A dedicated consideration of HMIS data quality is warranted, given the study’s reliance on routinely collected surveillance data. First, reporting completeness varies across facilities and over time. Systematic gaps in HMIS verification, including missing records and inconsistent coding, have been documented across Rwandan districts [68]. Apparent increases in case counts may therefore partly reflect improvements in reporting coverage rather than genuine incidence rises. This is particularly relevant for interpreting the 2018 surge and the post-2021 escalation, where stepwise improvements in HMIS completeness cannot be ruled out as contributing factors. Second, a high proportion of diarrhea diagnoses in Rwanda’s HMIS are recorded as unspecified, masking pathogen-specific trends and limiting the granularity with which transmission dynamics can be attributed to specific etiological drivers, a concern echoed in earlier assessments of HMIS reliability in Rwanda and Ethiopia [68,69]. Third, the COVID-19-related reduction in health facility utilization in 2020 represents a reporting disruption rather than a true transmission decline, as discussed in the Results; models trained on such disrupted periods may underweight the true endemic baseline when forecasting forward. Fourth, sex-disaggregated analysis was not conducted, as HMIS diarrhea reporting is not consistently captured by sex, a data-quality gap that should be prioritized in future surveillance improvements.

Despite these limitations, this study provides a practical and immediately usable forecasting foundation for Rwanda’s routine planning. The dataset spans 11 consecutive years of nationally aggregated monthly data with no missing observations. It is routinely audited at the facility level under Ministry of Health authorization and supervision. This level of temporal completeness and institutional oversight supports the reliability of the time-series analysis. The consistent age-stratified reporting architecture directly enabled the age-stratified SARIMA framework applied here, and the strong, reproducible seasonal signal corroborated by DHS prevalence trends further supports the internal validity of the findings. By applying transparent, reproducible Box–Jenkins methods with cross-validation and residual diagnostics, the approach is feasible to operationalize within routine analytics workflows [46,47,48,49], and moves beyond prediction alone by contextualizing forecasts within a provisional seasonal preparedness framework and proposing evidence-based exceedance signals that can inform pre-peak readiness and timely response. Building on this baseline, future work should benchmark SARIMA against more flexible approaches, including gradient boosting models that have demonstrated improved performance in Rwandan public health applications [70]. In parallel, hybrid strategies combining SARIMA with covariate-informed or Bayesian/ensemble methods should be developed to better adapt to evolving transmission dynamics and data quality shifts.

## 5. Conclusions

This study developed and validated age-stratified SARIMA models using Rwanda’s national HMIS data (2015–2025). The models characterized seasonal diarrheal dynamics and generated short- to medium-term case forecasts for 2026–2027, stratified by age group (under-five and five-and-above). Rwanda experienced a sustained and increasing diarrheal burden over the study period, with nearly half of all reported cases occurring among children under five, and incidence rates consistently several-fold higher in this group than in older ages. Both age strata exhibited a strong, recurrent annual seasonal cycle, and the selected SARIMA models captured this structure, forecasting that a substantial caseload will persist through 2026–2027.

Programmatically, the observed seasonal regularity and age-stratified burden patterns suggest a provisional preparedness framework, including a recurring late-dry-season peak window for commodity pre-positioning and surge readiness, and a distinction between population-driven absolute caseload in older age groups and the disproportionately higher per-child risk in under-fives. These implications are grounded in historical pattern-based evidence rather than causal modeling. They should be treated as indicative planning signals, contingent on regular forecast updates, rather than prescriptive operational mandates. Overall, this study demonstrates the feasibility of integrating SARIMA-based forecasting into routine HMIS surveillance workflows in Rwanda. Future work should focus on strengthening diagnostic specificity and incorporating external drivers or hybrid methods to improve responsiveness to structural shocks and evolving data quality.

## Figures and Tables

**Table 1:** Annual summary of diarrhea cases and incidence rates, 2015–2025.

Year	Under Five			Five and Above		
	Total Cases	Population	Incidence Rate (per 1000 People)	Total Cases	Population	Incidence Rate (per 1000 People)
2015	155,939	1,553,879	100.35	178,926	9,504,642	18.83
2016	148,834	1,622,775	91.72	178,971	9,954,236	17.98
2017	170,283	1,617,444	105.28	217,574	9,956,441	21.85
2018	382,059	1,664,629	229.52	217,827	10,251,200	21.25
2019	334,801	1,718,592	194.81	270,656	10,650,000	25.41
2020	271,043	1,744,458	155.37	273,735	10,857,340	25.21
2021	298,797	1,789,696	166.95	316,527	11,192,150	28.28
2022	326,144	1,709,387	190.80	396,138	11,544,200	34.31
2023	324,440	1,723,382	188.26	398,900	11,782,740	33.85
2024	362,560	1,750,005	207.18	381,940	12,054,750	31.68
2025	328,477	1,778,910	184.65	374,527	12,326,059	30.38

Note: Incidence Rate (Annual cumulative incidence per 1000 population) = (Total annual cases/mid-year population estimate) × 1000.

**Table 2:** Cross-validated final SARIMA model specifications and benchmark comparison, by age group.

Age Group	Model	CV RMSE	CV MAE	AIC	BIC	CV MAPE (%)
Under 5	Seasonal naïve	7042	5253	-	-	21.1
	ETS (auto)	5259	4031	-	-	15.7
	STL + drift	4616	3837	-	-	15.7
	**SARIMA(2,1,0)(1,0,1)[12] ^†^**	**1868.2**	**1629.4**	**1997.9**	**2011.1**	**6.7**
5 and Above	Seasonal naïve	3865	3319	-	-	13.8
	ETS (auto)	4331	3810	-	-	16.1
	STL + drift	2320	1927	-	-	7.55 *
	**SARIMA(1,1,0)(1,0,1)[12] ^†^**	**2186.9**	**1896.3**	**1929.6**	**1940.1**	**9.1**

Abbreviations: AIC = Akaike Information Criterion; BIC = Bayesian Information Criterion; CV = Cross-Validation; ETS = Exponential Smoothing State-Space; MAE = Mean Absolute Error; MAPE = Mean Absolute Percentage Error; RMSE = Root Mean Square Error; STL = Seasonal-Trend Decomposition using Loess. ^†^ Selected SARIMA specification (bold); AIC and BIC reported only for SARIMA models, as these criteria are not comparable across model families. * Lower MAPE value for STL + drift compared to SARIMA.

**Table 3:** SARIMA model parameter estimates by age group.

Age Group	Model	Parameter	Estimate	Standard Error	Z Statistic	*p* Value
Under 5	SARIMA (2,1,0)(1,0,1)[12]	AR1	0.8677	0.0493	17.596	<0.001
		AR2	–0.4421	0.0817	–5.411	<0.001
		SAR1	0.7679	0.1815	4.231	<0.001
		SMA1	–0.5307	0.239	–2.22	0.026
5 and Above	SARIMA (1,1,0)(1,0,1)[12]	AR1	–0.2158	0.0861	–2.508	0.012
		SAR1	0.9249	0.0684	13.529	<0.001
		SMA1	–0.6495	0.16	–4.059	<0.001

Abbreviations: AR = autoregressive; SAR = seasonal autoregressive; SMA = seasonal moving average.

**Table 4:** Annual forecast summary of diarrhea cases (2026–2027) by age group.

Year	Under Five		Five and Above	
	Annual Total	Monthly Mean (95% PI)	Annual Total	Monthly Mean (95% PI)
2026	354,439	29,537 (14,514–44,559)	405,616	33,801 (22,816–44,786)
2027	359,474	29,956 (22,512–58,036)	417,524	34,794 (14,089–55,498)

Note: 95% PI widen with forecast horizon as uncertainty compounds over time, a standard property of SARIMA models, not a model inconsistency.

## Data Availability

Restrictions apply to the availability of these data. Data were obtained from Rwanda’s Health Management Information System (HMIS) with permission from the Ministry of Health and are not publicly available. Aggregated data may be made available from the corresponding author upon reasonable request and with authorization from the Ministry of Health.

## Abbreviations

The following abbreviations are used in this manuscript:

ADF	Augmented Dickey–Fuller (test)
AIC	Akaike Information Criterion
AR	Autoregressive
ARIMA	Autoregressive Integrated Moving Average
BIC	Bayesian Information Criterion
CV	Cross-Validation
ETS	Exponential Smoothing State-Space
HMIS	Health Management Information System
MA	Moving Average
MAE	Mean Absolute Error
MAPE	Mean Absolute Percentage Error
RMSE	Root Mean Square Error
SARIMA	Seasonal Autoregressive Integrated Moving Average
STL	Seasonal-Trend decomposition using Loess

## References

[1] WHO Diarrhoeal Disease. https://www.who.int/news-room/fact-sheets/detail/diarrhoeal-disease#:~:text=Key%20facts,aged%205%20to%209%20years.

[2] Kyu H.H., Vongpradith A., Dominguez R.-M.V., Ma J., Albertson S.B., Novotney A., Khalil I.A., Troeger C.E., Doxey M.C., Ledesma J.R. (2025). Global, regional, and national age-sex-specific burden of diarrhoeal diseases, their risk factors, and aetiologies, 1990–2021, for 204 countries and territories: A systematic analysis for the Global Burden of Disease Study 2021. Lancet Infect. Dis..

[3] Claudine U., Kim J.Y., Kim E.M., Yong T.S. (2021). Association between Sociodemographic Factors and Diarrhea in Children Under 5 Years in Rwanda. Korean J. Parasitol..

[4] Troeger C., Blacker B.F., Khalil I.A., Rao P.C., Cao S., Zimsen S.R.M., Albertson S.B., Stanaway J.D., Deshpande A., Abebe Z. (2018). Estimates of the global, regional, and national morbidity, mortality, and aetiologies of diarrhoea in 195 countries: A systematic analysis for the Global Burden of Disease Study 2016. Lancet Infect. Dis..

[5] Thystrup C., Majowicz S.E., Kitila D.B., Desta B.N., Fayemi O.E., Ayolabi C.I., Hugho E., Buys E.M., Akanni G.B., Machava N.E. (2024). Etiology-specific incidence and mortality of diarrheal diseases in the African region: A systematic review and meta-analysis. BMC Public Health.

[6] NISR, MOH, ICF (2015). Rwanda Demographic and Health Survey, 2014–2015.

[7] NISR, MOH, ICF (2021). Rwanda Demographic and Health Survey 2019-20 Final Report.

[8] NISR, MOH, ICF (2025). Rwanda Demographic and Health Survey 2025, Key Indicators Report.

[9] Tareke A.A., Jemal S.S., Yemane G.D., Zakaria H.F., Shiferaw E.W., Ngabonzima A. (2024). Spatial disparity and associated factors of diarrhea among under-five children in Rwanda: A multilevel logistic regression analysis. BMC Pediatr..

[10] Wang Q., Li S., Li R. (2018). Forecasting energy demand in China and India: Using single-linear, hybrid-linear, and non-linear time series forecast techniques. Energy.

[11] Li S., Li R. (2017). Comparison of Forecasting Energy Consumption in Shandong, China Using the ARIMA Model, GM Model, and ARIMA-GM Model. Sustainability.

[12] Wang Q., Li S., Jiang F. (2021). Uncovering the impact of the COVID-19 pandemic on energy consumption: New insight from difference between pandemic-free scenario and actual electricity consumption in China. J. Clean. Prod..

[13] Wang G., Han Y., Chang J. (2024). Research on soil moisture content combination prediction model based on ARIMA and BP neural networks. Adv. Control Appl..

[14] Lu S. (2021). Research on GDP Forecast Analysis Combining BP Neural Network and ARIMA Model. Comput. Intell. Neurosci..

[15] Hauer M.E. (2019). Population projections for U.S. counties by age, sex, and race controlled to shared socioeconomic pathway. Sci. Data.

[16] ArunKumar K.E., Kalaga D.V., Sai Kumar C.M., Chilkoor G., Kawaji M., Brenza T.M. (2021). Forecasting the dynamics of cumulative COVID-19 cases (confirmed, recovered and deaths) for top-16 countries using statistical machine learning models: Auto-Regressive Integrated Moving Average (ARIMA) and Seasonal Auto-Regressive Integrated Moving Average (SARIMA). Appl. Soft Comput..

[17] Ceylan Z. (2020). Estimation of COVID-19 prevalence in Italy, Spain, and France. Sci. Total Environ..

[18] Roy S., Bhunia G.S., Shit P.K. (2021). Spatial prediction of COVID-19 epidemic using ARIMA techniques in India. Model. Earth Syst. Environ..

[19] Zhang X., Liu Y., Yang M., Zhang T., Young A.A., Li X. (2013). Comparative Study of Four Time Series Methods in Forecasting Typhoid Fever Incidence in China. PLoS ONE.

[20] Siamba S., Otieno A., Koech J. (2023). Application of ARIMA, and hybrid ARIMA Models in predicting and forecasting tuberculosis incidences among children in Homa Bay and Turkana Counties, Kenya. PLoS Digit. Health.

[21] Wei W., Jiang J., Gao L., Liang B., Huang J., Zang N., Ning C., Liao Y., Lai J., Yu J. (2017). A New Hybrid Model Using an Autoregressive Integrated Moving Average and a Generalized Regression Neural Network for the Incidence of Tuberculosis in Heng County, China. Am. J. Trop. Med. Hyg..

[22] Wang M., Pan J., Li X., Li M., Liu Z., Zhao Q., Luo L., Chen H., Chen S., Jiang F. (2022). ARIMA and ARIMA-ERNN models for prediction of pertussis incidence in mainland China from 2004 to 2021. BMC Public Health.

[23] Zhai M., Li W., Tie P., Wang X., Xie T., Ren H., Zhang Z., Song W., Quan D., Li M. (2021). Research on the predictive effect of a combined model of ARIMA and neural networks on human brucellosis in Shanxi Province, China: A time series predictive analysis. BMC Infect. Dis..

[24] Wu W., An S.-Y., Guan P., Huang D.-S., Zhou B.-S. (2019). Time series analysis of human brucellosis in mainland China by using Elman and Jordan recurrent neural networks. BMC Infect. Dis..

[25] Wang Y.W., Shen Z.Z., Jiang Y. (2018). Comparison of ARIMA and GM(1,1) models for prediction of hepatitis B in China. PLoS ONE.

[26] Jian Y., Zhu D., Zhou D., Li N., Du H., Dong X., Fu X., Tao D., Han B. (2022). ARIMA model for predicting chronic kidney disease and estimating its economic burden in China. BMC Public Health.

[27] Xian X., Wang L., Wu X., Tang X., Zhai X., Yu R., Qu L., Ye M. (2023). Comparison of SARIMA model, Holt-winters model and ETS model in predicting the incidence of foodborne disease. BMC Infect. Dis..

[28] Tosepu R., Ningsi N.Y. (2024). Forecasting of diarrhea disease using ARIMA model in Kendari City, Southeast Sulawesi Province, Indonesia. Heliyon.

[29] Arifin H., Rakhmawati W., Kurniawati Y., Pradipta R.O., Efendi F., Gusmaniarti G., Pramukti I., Acob J.R.U., Soares A., Myint N.M.M. (2022). Prevalence and determinants of diarrhea among under-five children in five Southeast Asian countries: Evidence from the demographic health survey. J. Pediatr. Nurs..

[30] Demissie G.D., Yeshaw Y., Aleminew W., Akalu Y. (2021). Diarrhea and associated factors among under five children in sub-Saharan Africa: Evidence from demographic and health surveys of 34 sub-Saharan countries. PLoS ONE.

[31] Brintz B.J., Howard J.I., Haaland B., Platts-Mills J.A., Greene T., Levine A.C., Nelson E.J., Pavia A.T., Kotloff K.L., Leung D.T. (2020). Clinical predictors for etiology of acute diarrhea in children in resource-limited settings. PLoS Negl. Trop. Dis..

[32] Paul P. (2020). Socio-demographic and environmental factors associated with diarrhoeal disease among children under five in India. BMC Public Health.

[33] Zemariam A.B., Abey W., Kassaw A.K., Yimer A. (2024). Comparative analysis of machine learning algorithms for predicting diarrhea among under-five children in Ethiopia: Evidence from 2016 EDHS. Health Inform. J..

[34] Ogwel B., Mzazi V.H., Awuor A.O., Okonji C., Anyango R.O., Oreso C., Ochieng J.B., Munga S., Nasrin D., Tickell K.D. (2025). Derivation and validation of a clinical predictive model for longer duration diarrhea among pediatric patients in Kenya using machine learning algorithms. BMC Med. Inform. Decis. Mak..

[35] Ashaolu J.O., Do L.T., Isaac K.R., Akanji T.S., Some S.Y.M. (2025). Determinants of childhood diarrhea in low- and middle-income countries: A comparative analysis of epidemiological and machine learning approaches. BMC Public Health.

[36] Yehuala T.Z., Derseh N.M., Tewelgne M.F., Wubante S.M. (2024). Exploring Machine Learning Algorithms to Predict Diarrhea Disease and Identify its Determinants among Under-Five Years Children in East Africa. J. Epidemiol. Glob. Health.

[37] Fang X., Liu W., Ai J., He M., Wu Y., Shi Y., Shen W., Bao C. (2020). Forecasting incidence of infectious diarrhea using random forest in Jiangsu Province, China. BMC Infect. Dis..

[38] Liang D., Wang L., Liu S., Li S., Zhou X., Xiao Y., Zhong P., Chen Y., Wang C., Xu S. (2024). Global Incidence of Diarrheal Diseases-An Update Using an Interpretable Predictive Model Based on XGBoost and SHAP: A Systematic Analysis. Nutrients.

[39] Wang P., Zhang W., Wang H., Shi C., Li Z., Wang D., Luo L., Du Z., Hao Y. (2024). Predicting the incidence of infectious diarrhea with symptom surveillance data using a stacking-based ensembled model. BMC Infect. Dis..

[40] Ogwel B., Mzazi V., Nyawanda B.O., Otieno G., Omore R. (2024). Predictive modeling for infectious diarrheal disease in pediatric populations: A systematic review. Learn. Health Syst..

[41] Luo Z., Jia X., Bao J., Song Z., Zhu H., Liu M., Yang Y., Shi X. (2022). A Combined Model of SARIMA and Prophet Models in Forecasting AIDS Incidence in Henan Province, China. Int. J. Environ. Res. Public Health.

[42] Zhao Z., Zhai M., Li G., Gao X., Song W., Wang X., Ren H., Cui Y., Qiao Y., Ren J. (2023). Study on the prediction effect of a combined model of SARIMA and LSTM based on SSA for influenza in Shanxi Province, China. BMC Infect. Dis..

[43] Waliullah M., Hossain M.J., Hasan M.R., Hannan A., Rahman M.M. (2025). Unveiling the future: Wavelet-ARIMAX analysis of climate and diarrhea dynamics in Bangladesh’s Urban centers. BMC Public Health.

[44] NISR (2023). The Fifth Rwanda Population and Housing Census, Thematic Report: Population Projections.

[45] WHO (2005). The Treatment of Diarrhoea: A Manual for Physicians and Other Senior Health Workers.

[46] Box G.E., Jenkins G.M., Reinsel G.C., Ljung G.M. (2015). Time Series Analysis: Forecasting and Control.

[47] Hyndman R.J., Athanasopoulos G. (2021). Forecasting: Principles and Practice.

[48] Dickey D.A., Fuller W.A. (1979). Distribution of the Estimators for Autoregressive Time Series with a Unit Root. J. Am. Stat. Assoc..

[49] Ljung G.M., Box G.E.P. (1978). On a Measure of Lack of Fit in Time Series Models. Biometrika.

[50] Wanyana D., Wong R., Hakizimana D. (2021). Rapid assessment on the utilization of maternal and child health services during COVID-19 in Rwanda. Public Health Action.

[51] Butera E., Habimana J.D., Korukire N., Nsereko E., Xavier S.F., Nikwigize S., Michoel H.F. (2025). Trends and Risk Factors For Childhood Diarrhoea in Rwanda: A Secondary Data Analysis of Three Rwanda Demographic and Health Surveys (RDHS 2010, 2014/15, and 2019/20). Rwanda J. Med. Health Sci..

[52] Alexander K.A., Heaney A.K., Shaman J. (2018). Hydrometeorology and flood pulse dynamics drive diarrheal disease outbreaks and increase vulnerability to climate change in surface-water-dependent populations: A retrospective analysis. PLoS Med..

[53] Deshpande A., Chang H.H., Levy K. (2020). Heavy Rainfall Events and Diarrheal Diseases: The Role of Urban-Rural Geography. Am. J. Trop. Med. Hyg..

[54] Wu J., Yunus M., Streatfield P.K., Emch M. (2014). Association of climate variability and childhood diarrhoeal disease in rural Bangladesh, 2000–2006. Epidemiol. Infect..

[55] Lee T.T., Dalvie M.A., Röösli M., Merten S., Kwiatkowski M., Mahomed H., Sweijd N., Cissé G. (2023). Understanding diarrhoeal diseases in response to climate variability and drought in Cape Town, South Africa: A mixed methods approach. Infect. Dis. Poverty.

[56] Aik J., Ong J., Ng L.-C. (2020). The effects of climate variability and seasonal influence on diarrhoeal disease in the tropical city-state of Singapore—A time-series analysis. Int. J. Hyg. Environ. Health.

[57] Perone G. (2022). Using the SARIMA Model to Forecast the Fourth Global Wave of Cumulative Deaths from COVID-19: Evidence from 12 Hard-Hit Big Countries. Econometrics.

[58] Cong J., Ren M., Xie S., Wang P. (2019). Predicting Seasonal Influenza Based on SARIMA Model, in Mainland China from 2005 to 2018. Int. J. Environ. Res. Public Health.

[59] Liu J., Yu F., Song H. (2023). Application of SARIMA model in forecasting and analyzing inpatient cases of acute mountain sickness. BMC Public Health.

[60] Azanaw J., Malede A., Yalew H.F., Worede E.A. (2024). Determinants of diarrhoeal diseases among under-five children in Africa (2013–2023): A comprehensive systematic review highlighting geographic variances, socioeconomic influences, and environmental factors. BMC Public Health.

[61] Desai A.N., Kraemer M.U.G., Bhatia S., Cori A., Nouvellet P., Herringer M., Cohn E.L., Carrion M., Brownstein J.S., Madoff L.C. (2019). Real-time Epidemic Forecasting: Challenges and Opportunities. Health Secur..

[62] Gnanvi J.E., Salako K.V., Kotanmi G.B., Glèlè Kakaï R. (2021). On the reliability of predictions on COVID-19 dynamics: A systematic and critical review of modelling techniques. Infect. Dis. Model..

[63] Armando C.J., Rocklöv J., Sidat M., Tozan Y., Mavume A.F., Sewe M.O. (2025). Spatio-temporal modelling and prediction of malaria incidence in Mozambique using climatic indicators from 2001 to 2018. Sci. Rep..

[64] Jiao S., Wang Y., Ye X., Nagahara L., Sakurai T. (2025). Spatio-temporal epidemic forecasting using mobility data with LSTM networks and attention mechanism. Sci. Rep..

[65] Semakula M., Niragire F., Faes C. (2023). Spatio-Temporal Bayesian Models for Malaria Risk Using Survey and Health Facility Routine Data in Rwanda. Int. J. Environ. Res. Public Health.

[66] Nixon K., Jindal S., Parker F., Marshall M., Reich N.G., Ghobadi K., Lee E.C., Truelove S., Gardner L. (2022). Real-time COVID-19 forecasting: Challenges and opportunities of model performance and translation. Lancet Digit. Health.

[67] Karasinghe N., Peiris S., Jayathilaka R., Dharmasena T. (2024). Forecasting weekly dengue incidence in Sri Lanka: Modified Autoregressive Integrated Moving Average modeling approach. PLoS ONE.

[68] Nshimyiryo A., Kirk C.M., Sauer S.M., Ntawuyirusha E., Muhire A., Sayinzoga F., Hedt-Gauthier B. (2020). Health management information system (HMIS) data verification: A case study in four districts in Rwanda. PLoS ONE.

[69] Ouedraogo M., Kurji J., Abebe L., Labonté R., Morankar S., Bedru K.H., Bulcha G., Abera M., Potter B.K., Roy-Gagnon M.H. (2019). A quality assessment of Health Management Information System (HMIS) data for maternal and child health in Jimma Zone, Ethiopia. PLoS ONE.

[70] Ndagijimana S., Kabano I.H., Masabo E., Ntaganda J.M. (2023). Prediction of Stunting Among Under-5 Children in Rwanda Using Machine Learning Techniques. J. Prev. Med. Public Health.

